# The Effect of Salt Intake and Potassium Supplementation on Serum Gastrin Levels in Chinese Adults: A Randomized Trial

**DOI:** 10.3390/nu9040389

**Published:** 2017-04-14

**Authors:** Yuan-Yuan Wang, Wen-Wen He, Yan-Chun Liu, Yi-Feng Lin, Lu-Fei Hong

**Affiliations:** 1 Department of Physics, Harbin University, Harbin 150086, China; gongxlyc@hrbu.edu.cn; 2 Cardio-Cerebrovascular Control and Research Center, Institute of Basic Medicine, Shandong Academy of Medical Sciences, Jinan 250062, China; hewenwenxxk@hrbu.edu.cn (W.-W.H.); linyf1990@hrbu.edu.cn (Y.-F.L.)

**Keywords:** gastrin, salt, potassium, sodium excretion, blood pressure

## Abstract

Excess dietary salt is strongly correlated with cardiovascular disease, morbidity, and mortality. Conversely, potassium likely elicits favorable effects against cardiovascular disorders. Gastrin, which is produced by the G-cells of the stomach and duodenum, can increase renal sodium excretion and regulate blood pressure by acting on the cholecystokinin B receptor. The aim of our study was to assess the effects of altered salt and potassium supplementation on serum gastrin levels in humans. A total of 44 subjects (38–65 years old) were selected from a rural community in northern China. All subjects were sequentially maintained on a relatively low-salt diet for 7 days (3.0 g/day of NaCl), a high-salt diet for 7 days (18.0 g/day of NaCl), and then a high-salt diet supplemented with potassium for another 7 days (18.0 g/day of NaCl + 4.5 g/day of KCl). The high-salt intake significantly increased serum gastrin levels (15.3 ± 0.3 vs. 17.6 ± 0.3 pmol/L). This phenomenon was alleviated through potassium supplementation (17.6 ± 0.3 vs. 16.5 ± 0.4 pmol/L). Further analyses revealed that serum gastrin was positively correlated with 24 h urinary sodium excretion (*r* = 0.476, *p* < 0.001). By contrast, gastrin level was negatively correlated with blood pressure in all dietary interventions (*r* = −0.188, *p* = 0.031). The present study indicated that variations in dietary salt and potassium supplementation affected the serum gastrin concentrations in the Chinese subjects.

## 1. Introduction

Excess dietary salt induces adverse cardiovascular and renal effects according to epidemiological, interventional, and experimental studies [1,2,3]. In addition, hypertension prevention studies have proven that moderate salt reduction promotes 25% long-term reduction in the risk of cardiovascular events; thus, salt intake is related to cardiovascular function [4]. Several mechanisms, including endothelial dysfunction, oxidative stress, inflammation, insulin resistance, and neurogenically mediated increase in peripheral resistance, contribute to the harmful effects of dietary salt [5,6,7,8]. 

Gastrin, a peptide hormone secreted primarily by G-cells in response to food ingestion, is released into the blood stream. It is the principal mediator of meal-induced gastric phase and acid secretion and acts via paracrine stimulation of histamine released from gastric enterochromaffin-like cells [9,10,11]. Gastrin acts on its receptor, cholecystokinin B receptor (CCKBR), to regulate gastric acid secretion and oxyntic gland proliferation and exert physiological actions outside the gastric mucosa, particularly in the colon, pancreas, small intestine, liver, esophagus, and kidney [12]. Furthermore, CCKBR is widely expressed in the kidney, especially in the glomerular mesangial cells, collecting duct cells, and proximal convoluted tubule cells [13,14]. Gastrin also regulates sodium balance and blood pressure [10,15]. Notably, natriuresis of a certain amount of Na^+^ after ingestion may be caused by gastrin, which is taken up by renal cortical tubules to a greater extent than the other enterokines released [16]. However, the relationship between circulatory gastrin levels and dietary salt intake in humans has not been elucidated. 

Potassium intake likely elicits favorable effects on cardiovascular morbidity and mortality. Dietary potassium supplementation remarkably alleviates blood pressure (BP) increase induced by high amounts of dietary salt through the inhibition of sympathetic nerve excitability, salt-induced insulin resistance, and oxygen-reactive free radical generation [17,18]. Long-term potassium supplementation may also lower the risk of cardiovascular disease [19], and high potassium intake benefits arterial compliance and stiffness [20]. However, data on the link between potassium supplementation and serum gastrin levels are sparse.

In this study, we prospectively examined the effects of salt and potassium supplementation on serum gastrin levels in normotensive and mildly hypertensive subjects. Moreover, the correlations among serum gastrin, BP, and urinary sodium excretion were investigated.

## 2. Materials and Methods

### 2.1. Subjects

After screening subjects with similar dietary habits and from a rural community in northern China, 44 of the 49 subjects were enrolled in this study. Five subjects were excluded because of diabetes, kidney disease or unwilling participation. The sample size was calculated based on the frequency of evacuation, and the standard deviation of the difference was 0.8 between periods [21]. Therefore, a total sample size of 44 was sufficient to expect a 95% power with a two-sided significance level of 0.05. Moreover, a brief medical questionnaire was administered. Subjects were considered to have hypertension if they had a mean systolic BP (SBP) of ≥140 mmHg and/or a mean diastolic BP (DBP) of ≥90 mmHg, or if they were taking one or more antihypertensive medications. BP levels could increase after the dietary intervention; therefore, we excluded those with stage 2 hypertension. The other exclusion criteria were secondary hypertension; history of clinical cardiovascular disease, chronic liver and kidney disease, or diabetes; use of antihypertensive medication; pregnancy; and high alcohol intake.

The research was approved by the institutional ethics committee of the Institute of Basic Medicine, Shandong Academy of Medical Sciences (Code: SAMS2015-034) according to the Declaration of Helsinki (2008) of the World Medical Association. All participants provided written informed consent.

### 2.2. Dietary Intervention

The chronic salt intake and potassium supplementation intervention protocol was performed as described elsewhere [22,23]. The protocol consisted of a series of investigations, including a 3-day baseline period, during which clinical history and physical examination data (height, weight, and BP) were obtained. After the 3-day baseline observations, the study participants received a 7-day salt (NaCl) intake of 3.0 g/day, followed by a 7-day salt intake of 18.0 g/day. Dietary potassium intake remained unchanged during the first two intervention phases. In the final week, the participants maintained the high-salt diet and took a 60 mmol potassium (KCl) supplement. All study foods were cooked without salt, and prepackaged salt was added to each meal of a study participant. The participants were required to have their breakfast, lunch, and dinner in the study kitchen under the supervision of the study staff throughout the intervention period, in order to ensure compliance with the intervention program. The study participants were also instructed to avoid consuming foods that were not provided by the study personnel. 

### 2.3. BP Measurement

BP levels were measured by three trained staff members using a standard mercury sphygmomanometer. The measurements were performed while the subjects were in a sitting position after they had rested for ≥5 min. For each patient, BP was measured three times at 1 min intervals during the 3-day baseline observation period, and on days 6 and 7 of each of the three 7-day intervention periods. BP observers were blinded to the participants’ dietary interventions. The subjects were instructed to avoid alcohol, cigarette smoking, coffee/tea, and exercise for at least 30 min prior to the BP measurements. The SBP and DBP were determined through the first and fifth Korotkoff sounds, respectively. The pulse pressure was calculated as SBP—DBP. The mean arterial pressure (MAP) was calculated as DBP + (1/3 × pulse pressure). The BP at baseline and during the intervention was calculated as the mean of six measurements from two clinical visits during the 3-day baseline observation period and the mean of the measurements on days 6 and 7 of each of the three 7-day intervention periods, respectively.

### 2.4. Biochemical Analyses

Fasting blood samples were obtained on the last day of each intervention period through peripheral venous puncture, immediately centrifuged at 3000× *g* for 10 min, and stored at −80 °C until analysis. The total cholesterol, triglyceride, low-density lipoprotein cholesterol (LDL-C), high-density lipoprotein cholesterol (HDL-C), serum creatinine, and fasting plasma blood glucose levels were evaluated with an automatic biochemical analyzer (model 7600; Hitachi, Ltd., Tokyo, Japan). Serum gastrin levels were assessed by commercially available enzyme-linked immunosorbent assay (ELISA) kits (Uscn Life Science, Wuhan, China). Five urine samples were used to evaluate intra- and inter-assay coefficients of variation, which ranged from 2.1%–4.5% and 3.2%–7.4%, respectively, for gastrin.

### 2.5. 24 h Urinary Sodium, Potassium and Creatinine Determination

24-h urine samples were collected at baseline and on day 7 of each intervention period. Any urine collections less than 500 mL or with a creatinine excretion lower than the population mean minus two SDs were discarded to ensure the completeness of the collection [24]. The samples were kept frozen at −40 °C until analysis. Urinary concentrations of sodium, potassium and creatinine were determined with ion-selective electrodes (Hitachi, Ltd., Tokyo, Japan). The 24-h urinary excretions of sodium, potassium and creatinine were quantified by multiplying the sodium, potassium and creatinine concentrations by the 24-h total urine volume.

### 2.6. Statistical Analyses

Continuous data are presented as the mean ± standard error. Categorical data are expressed as frequencies with percentages. Differences in repeated measures were analyzed by repeated-measures analysis of variance. Pearson’s correlation coefficient was performed to determine the correlations if the residuals were normally distributed, and Spearman’s correlation coefficient was employed if otherwise. Statistical analyses were performed in SPSS for Windows, Version 16.0 (SPSS Inc., Chicago, IL, USA). A two-tailed *p*-value of < 0.05 was considered statistically significant.

## 3. Results

### 3.1. Profiles of Studied Subjects

All subjects completed the intervention trials. The demographic and clinical characteristics of the study participants are highlighted in Table 1. Five subjects (11.4%) had hypertension, and none of them were taking medication.

### 3.2. Effects of Salt Intake and Potassium Supplementation on BP and 24 h Urinary Sodium and Potassium Excretion

At baseline, the 24-h urinary sodium and potassium excretion averaged 173.7 ± 10.5 (approximately 10.2 g of salt per day, 1 g salt = 17.1 mmol Na) and 47.0 ± 3.0 mmol/day (approximately 1.83 g of K per day, 1 mmol = 39 mg K), respectively. The BP responses to the relatively low-salt, high-salt, and high-salt + potassium supplementation interventions are presented in Table 2. The BP levels significantly increased when the interventions changed from the relatively low- to high-salt diet and decreased when the intervention changed from the high-salt to high-salt + potassium supplementation (*p* < 0.05). 

The 24-h sodium and potassium excretions in the urine were calculated after each intervention period to ensure the compliance of the participants. As shown in Table 2, the urinary sodium excretion significantly decreased as the baseline diet changed to relatively low-salt, but increased when the relatively low-salt diet was changed to high-salt (*p* < 0.05). Potassium supplementation significantly increased the urinary potassium excretion. The 24-h urinary creatinine excretion remained unchanged during the baseline and three intervention periods (*p* > 0.05). The data showed that the compliance of the subjects with the dietary interventions was excellent. 

### 3.3. Effects of Salt Intake and Potassium Supplementation on Fasting Gastrin Levels

**Figure 1:**
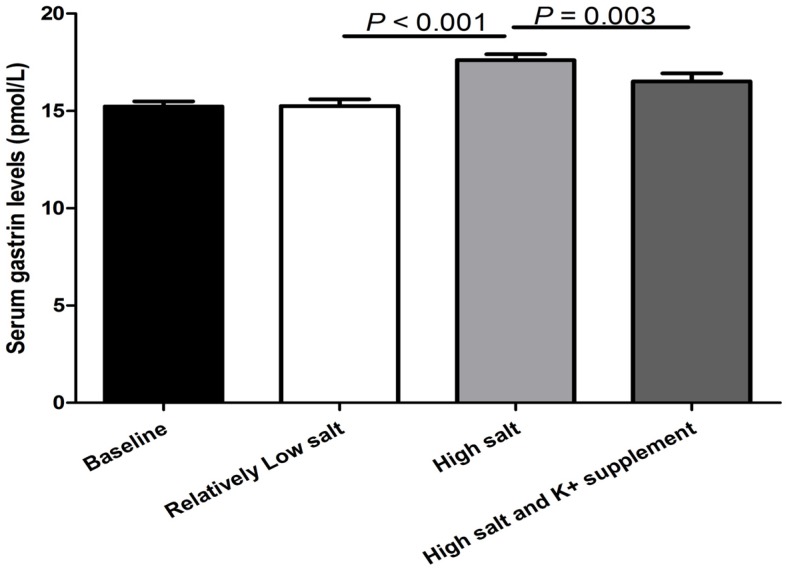
The effect of relatively low-salt, high-salt intake and potassium supplementation on serum gastrin levels in all subjects (*n* = 44).

The fasting gastrin levels were significantly increased with the change from the relatively low-salt diet to the high-salt diet (increased from 15.3 ± 0.3 to 17.6 ± 0.3 pmol/L, *p* < 0.001). The high-salt-diet-induced increase in serum gastrin was reduced by potassium supplementation (17.6 ± 0.3 vs. 16.5 ± 0.4 pmol/L, *p* = 0.003; Figure 1).

**Figure 2:**
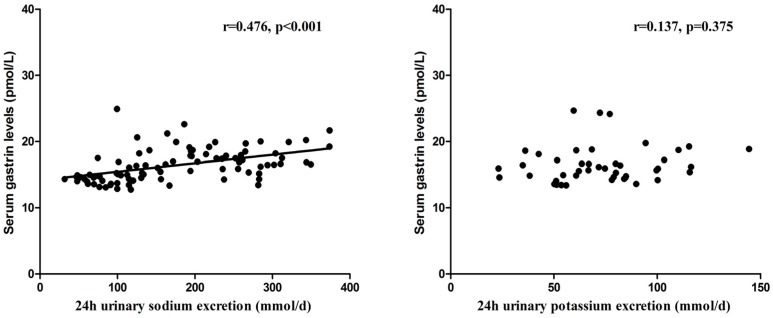
The correlation between serum gastrin levels and 24-h urinary sodium and potassium excretions in all subjects (*n* = 44) on a relatively low-salt diet, a high-salt diet, or on a high-salt diet with potassium supplementation.

**Figure 3:**
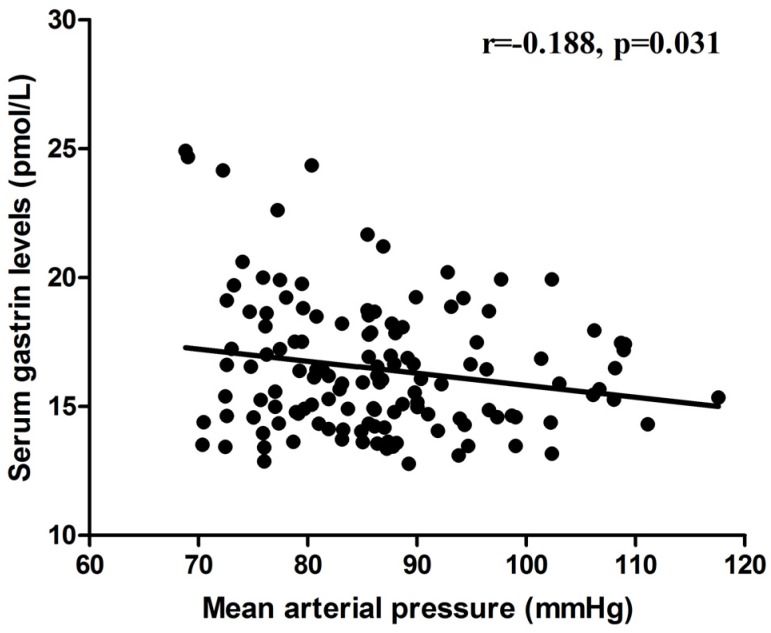
The correlation between serum gastrin and MAP in all subjects (*n* = 44) on a relatively low-salt diet, high-salt diet, and high-salt diet with potassium supplementation.

Further analyses revealed that the serum gastrin concentration was positively correlated with the 24-h urinary sodium excretion in the relatively low- and high-salt dietary intervention periods (*r* = 0.476, *p* < 0.001; Figure 2), but was not correlated with the 24-h urinary potassium excretion in the high-salt diet + potassium supplementation intervention period (*r* = 0.137, *p* = 0.375; Figure 2). Moreover, negative correlation was observed between the serum gastrin level and BP in the three intervention periods (*r* = −0.188, *p* = 0.031; Figure 3).

## 4. Discussion

The results of the present study demonstrate that high salt intake increases serum gastrin levels from the levels of the relatively low-salt diet. In addition, a positive correlation between the 24-h urinary sodium excretion and serum gastrin level is demonstrated in these Chinese participants. These data indicate that dietary salt intake significantly influences serum gastrin levels. 

Depending on the state of sodium balance, an oral NaCl load has been reported to produce stronger natriuresis and diuresis than an intravenous infusion of the same amount of NaCl, indicating the existence of a gastro-renal axis [9,25,26]. Several hormones secreted by the stomach and duodenum have been suggested to be effectors of the gastro-renal axis [9,10,25,26,27]. For example, an effector of the gastro-renal axis may be gastrin [9,10]. Food intake increases circulatory gastrin levels 10- to 20-fold more than those of cholecystokinin (CCK) [9,10,27]; furthermore, among the gut hormones, gastrin is the most absorbed hormone by the renal proximal tubules (RPTs) [16]. The existence of CCKBR in the gastrointestinal tract and the kidney suggests that gastrin, via CCKBR, may exert a coordinated regulation of sodium balance by regulating sodium transport in the gastrointestinal tract and kidney. Gastrin, via CCKBR, increases sodium excretion in the isolated perfused rat kidney [28]. Chen et al. [15] reported that the intrarenal infusion of gastrin increased sodium excretion and decreased Na^+^-K^+^-ATPase activity. In the present study, to exclude the effects of food intake on serum gastrin, we tested fasting blood samples collected at the last day of each period. We found that serum gastrin levels were markedly increased during high-salt intake and this finding was further reinforced by the observation that serum gastrin level positively correlated with urinary sodium excretion in humans. Therefore, high salt may promote natriuresis by increasing the levels of circulating gastrin, which is taken up by RPTs and inhibits Na^+^-K^+^-ATPase. However, the cellular mechanisms and signal transduction pathways by which gastrin regulates Na^+^-K^+^-ATPase activity are unknown. Liu et al. [29,30] showed that in immortalized human renal proximal tubule cells, gastrin, through CCKBR, inhibits sodium hydrogen exchanger type 3 (NHE3) and Na^+^-K^+^-ATPase activity via the phosphatidylinositol (PI) 3-kinase (PI3K)/protein kinase C (PKC) pathway. Future study investigating the additional mechanisms of this process would be very interesting.

Gastrin, produced by the G-cells of the stomach and duodenum, via CCKBR, expressed in several nephron segments, decreases Na^+^ transport and regulates blood pressure. Wank et al. [10] discovered that mice lacking gastrin (i.e., Gast^−/−^), systemically or only in the gut, or functional gastrin receptors (e.g., Cckbr^−/−^, CCKBR blockade) do not increase Na^+^ excretion after an oral Na^+^ load but have increased blood pressure. Genome-wide association studies showed that the chromosomal loci of gastrin and CCKBR were linked to hypertension [31,32]. Moreover, gastrin, upon interacting with dopamine, is involved in the normal regulation of renal sodium and blood pressure. Indeed, D1-like receptors, such as D1 dopamine receptor, and CCKBR synergistically increase sodium excretion in normotensive but not in spontaneously hypertensive rats, suggesting that the dysregulation of interaction between gastrin and CCKBR may be a mechanism in hypertension progress [15]. This hypothesis was supported by the study of Jiang et al. [33], who observed that the postgrandial gastrin levels were significantly higher in hypertensive adults than in normotensive adults, despite their similar fasting gastrin levels. In the current study, we observed a negative correlation between serum gastrin concentration and BP levels in the three intervention periods. In addition, gastrin is important in salt sensitivity, as indicated by result of the investigation on Gast^−/−^ mice, which are normotensive at a low Na^+^ intake but become hypertensive at a normal or high Na^+^ intake [34]. Determining the molecular mechanism and signaling molecules responsible for the effects of gastrin on salt sensitivity and hypertension could be of great interest.

The study represents, to our knowledge, the first report regarding the association between potassium intake and serum gastrin in the Chinese population. We found that potassium supplementation can reverse the effects of a high-salt diet on serum gastrin levels. The mechanism involved in the regulation of serum gastrin levels through potassium intake remains unclear, and thus requires further investigation. In the present study, potassium supplementation facilitates renal sodium excretion and thus may reduce the effects of high salt levels on gastrin levels. Furthermore, it is possible that potassium may directly influence gastrin activation, synthesis, and secretion. However, we found that the serum gastrin level was not correlated with urinary potassium excretion at the high-salt + potassium supplementation intervention. This effect may be due to the intervention method and individual differences among the study subjects.

The present study has some limitations. First, the effect of the low-salt diet does not seem very ideal, because the test subjects’ urinary excretions of sodium were found to be 101.2 mmol/day, which corresponds approximately to 5.9 g of salt. However, renal excretion of sodium is strictly regulated and affected by a variety of factors, in which dietary salt intake plays a very important role, but is not the only influencing factor. The individuals’ food intake, daily physical activity, and weather changes all can affect natriuresis to a certain extent. In addition, only one 24-h urinary collection was used to determine one’s dietary salt intake in our study. However, participation in the dietary interventions was high, and compliance with the study interventions, as assessed by urinary excretions of sodium and creatinine during each intervention period, was good. Furthermore, the study population is small and restricted to northern Chinese individuals. Therefore, our results require replication in other cohorts to determine generalizability to other ethnicities and populations with different backgrounds.

In summary, our human intervention study revealed that salt loading could increase circulating gastrin, and potassium supplementation could reverse the effects of excessive gastrin. Our findings indicate that the elevation of gastrin might be the underlying mechanism of salt-induced hypertension, which sheds some new light on prevention and a possible therapeutic target for hypertension in the future.

## Figures and Tables

**Table 1:** Baseline Demographic and Clinical Characteristics.

Parameters	Values
Age, year	51.8 ± 1.1
Sex (M/F)	21/23
Body mass index, kg/m^2^	23.5 ± 0.4
Alcohol (*n*, %)	4 (9.1)
Smoking (*n*, %)	19 (43.2)
Hypertension (*n*, %)	5 (11.4)
Systolic blood pressure, mmHg	110.4 ± 2.2
Diastolic blood pressure, mmHg	72.5 ± 1.3
Mean arterial pressure, mmHg	85.1 ± 1.5
Glucose, mmol/L	3.91 ± 0.10
Total cholesterol, mmol/L	4.11 ± 0.13
Triglycerides, mmol/L	1.24 ± 0.08
LDL-cholesterol, mmol/L	2.30 ± 0.11
HDL-cholesterol, mmol/L	1.20 ± 0.04
Serum creatinine, umol/L	56.6 ± 1.3

Values are means ± SE; LDL, low-density lipoprotein; HDL, high-density lipoprotein.

**Table 2:** BP Levels (mmHg) and 24-h Urinary Sodium, Potassium (mmol/day) and Creatinine (ìmol/day) Excretions at Baseline and During Dietary Interventions (*n* = 44).

Periods	SBP	DBP	24-h Urinary Na^+^	24-h Urinary K^+^	24-h Urinary Creatinine
Baseline	110.4 ± 2.2	72.5 ± 1.3	173.7 ± 10.5	47.0 ± 3.0	9002.7 ± 519.0
Relatively low salt	108.8 ± 1.9	73.8 ± 1.1	101.2 ± 5.8 ^§^	37.9 ± 2.9 ^§^	8248.2 ± 350.2
High salt	116.2 ± 2.7 *	76.9 ± 1.3 *	251.7 ± 9.3 *	42.3 ± 4.1	8462.1 ± 574.3
High salt and K^+^ supplement	107.3 ± 2.0 ^†^	71.9 ± 1.3 ^†^	266.0 ± 13.5	73.0 ± 4.0 ^†^	8839.8 ± 668.9

Values are means ± SE; ^§^ *p* < 0.05 vs. baseline. * *p* < 0.05 vs. relatively low-salt diet; ^†^ *p* < 0.05 vs. high-salt diet. BP, blood pressure; SBP, systolic blood pressure; DBP, diastolic blood pressure.
